# Post-pandemic Economics and Health Equity: Comment on "Ensuring Global Health Equity in a Post-pandemic Economy"

**DOI:** 10.34172/ijhpm.2023.7695

**Published:** 2023-02-26

**Authors:** Karime Chahuán-Jiménez

**Affiliations:** Escuela de Auditoría, Centro de Investigación en Negocios y Gestión Empresarial, Universidad de Valparaíso, Valparaíso, Chile.

**Keywords:** Post-pandemic Economics, Health Equity, Economic Theory, Conceptual Clusters, Sustainable Development

## Abstract

This article aims to compare the foundations of the post-pandemic economy and its impact on health equity, according to Labonté with the economics theory. The methodology developed is based on bibliometrics analysis, the documents, and specifications for a cluster of concepts, allowing deepened exposure of Labonté, complementing with the latest publications on the post-pandemic economy. Finally, the results agreed with Labonté about to economic development for achieving an economy that allows health equity considering sustainable development and the possibility of achieving the livelihood of Green New Deal as a basis.

## Introduction

 The pandemic of the SARS-CoV-2 health crisis declared by the World Health Organization (WHO) in January 2020 generated a rumble in world economies. The markets reflected the breakdown caused by the pandemic in March 2020^1^ due to border closures and quarantine warnings in different countries.

 As the pandemic progressed, different support programs were developed. However, the main one was the search for an alternative in the health area, the achievement of a vaccine to counteract severe and fatal effects on people.

 Once the vaccine was generated and massive vaccination processes began, countries began to move forward with openings because the economies had been strongly affected. Recovery was an inevitable challenge, Labonté^2^ indicates that among the proposals requested was a rapid transition to a ‘degrowth’ or ‘post-growth’ economy in which the world would be subjected to an extreme diet of material consumption, considering that the common to all proposals was mainly linked to the existing socioeconomic inequality.

 The commentary aims to compare the foundations of the post-pandemic economy and its impact from a health equity perspective, presented in Labonté’s *ensuring global health equity in a post-pandemic economy*, with the economic theory in the function of conceptual clusters.

 In the development of bibliometric analysis (Figure) associated with the generation of clusters with the mains concepts linked with the article: economy and post-pandemic, the clusters identified are those of climate change, economic growth, investment, politics (tax), globalization, and sustainable development, that are the topics on which this commentary was based, those clusters are also comparing with each section of Labonté’s manuscript and presented in Table.

**Figure:**
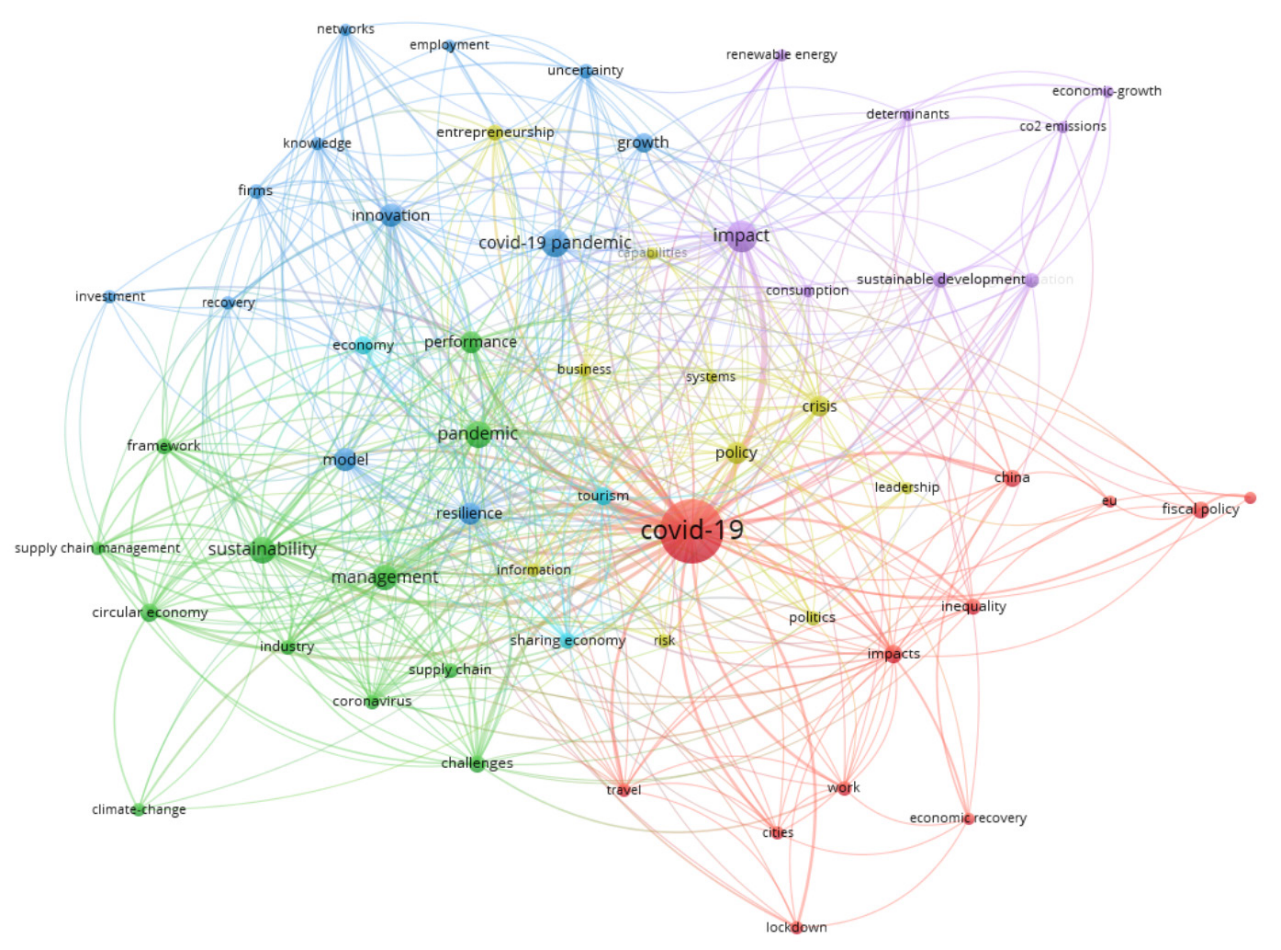
Bibliometric Analysis Based on the Post-COVID-19 Economy.

**Table:** Conceptual Cluster and the Relation With Explain of Labonté (2022)

**Bibliometric Analysis**	**Labonté (2022)**
Climate change	Degrowth/post-growth: Should we build back at all? Indicating that most of the responsibility lies with citizens, governments, and corporate actors in the historically over-consuming Global North.
Economic growth	Economic development, proposals based on the use of green energy.
Investment	From shareholder to stakeholder capitalism: More of the same?
Fiscal Policy	Tax and fiscal policy space: Can we build back fairer?
Globalization	Indications presented according to the World Economic Forum, proposal to build back better from the United States and other countries, and the European Union.
Sustainable development	Indicated in from shareholder capitalism to shareholder capitalism: More of the same? Incorporating the concept of degrowth from an environmental economy.
	Socio-economic inequality.

Table shows that only the topic of Labonté socio-economic inequality is not considered a conceptual cluster because the bibliometrics analysis has a global focus global more than a qualitative vision.

## Severe Acute Respiratory Syndrome Coronavirus 2

 More than two years after having decreed border closures and quarantines in different countries of the world (March 2020), today, the return to normality is a reality, considered a new normal, according to Labonté. One of the main reasons the world has been able to return to this new normal is vaccines, which have been applied to the extent that countries have been able to obtain them, depending on their capacity. Globally, an average of 80% have received at least one dose in North America, Latin America, and the Asia Pacific, an average of 69% in Europe and the Middle East, 57%, and in Africa, 27%.^3^ However, some consequences are inherited from SARS-CoV-2.

 This reflects the above-mentioned^1^ in which the growing disparities in wealth and power have undermined advances in health, also presented by Labonté, especially initially access to vaccination, since they required purchasing power for vaccines, as well as negotiating and accessing them, considering the conditions under which each of the countries is operating in a globalized world.

 COVID-19 intensified many economic and social problems societies were already facing,^4^ also inequality and climate change according to Labonté’s explanation.

## From Shareholder to Stakeholder Capitalism: More of the Same?

 Growth-driven natural capitalism supports its key contradictions.^5^ Its fundamental conflicts can be focused on cultural politics and political economy, which by 2008 were already defending the environment, and focusing on climate change. Labonté^2^ presents the elements of neoliberalism based, trade and financial liberalization, low taxes, minimal state intervention, and substantial property rights, which gave rise to our now familiar globalized economy, which were criticized almost from the beginning because of the inequalities it was promoting. However, it is indicated that the problem lies in maximizing profits for investors from an economic point of view since capitalism involves shareholders and all interested parties. Members of civil society also exert pressure on themselves, ensuring that no individual interest prevails over other interests.^6^ The Stakeholder theory cannot replace a theory of civil society developed in the 19th century.

 Labonté´s proposal is relevant because it gives way to the economic explanation for a focus of attention for new capitalism is that of sustainable capitalism, that is, on maximizing profit for all interested parties involved in achieving sustainable development, including the Sustainable Development Goals (SDGs) in developing business and resource generation activities. In addition, the SDGs must be included in economic development, accompanied by sustainable finance and adapting the principles of sustainable development for financial intermediaries.^7^ Therefore, the model of sustainable stakeholder capitalism will collaborate in the redistribution of wealth; however, it will strengthen the role of the private sector (not selfless and growing) in global health governance.^8^

## The Return of the State: Can Governments Mitigate the Inequalism Inherent in Capitalism?

 Globally, the COVID-19 pandemic has affected more than 214 countries worldwide, creating uncertainty and affecting all institutions and people.^9^ Implementing agile projects is the key to survival in the post-pandemic situation, but emerging economies have a limited scope for implementation. Organizations recognize the need for agile projects that can offer several benefits, such as faster implementation, adaptability, and better alignment to meet customer needs.

 A notable fact in governance is that the principles of new governance, such as the value of money and the pluralization of service delivery, are being put aside when governments urgently need to stop the spread of infection.^10^

 The post-pandemic recovery plans for those countries with the required fiscal capacities seemed to embody that transformative, both micro and macro-economic optimism,^11^ according to Labonté. The Green New Deal still promises substantial environmental protection, a rapid change in fossil fuels, and new expansionary social spending, as in the case of the European Union. Their support corresponds to the implementation of numerous recovery plans for Member States to try to mitigate the damage caused by COVID-19. The most critical element of this program is the Recovery and Resilience Mechanism, endowed with 672.5 billion euros in loans and grants. Seventy percent of Recovery and Resilience Facility grants will be distributed between 2021 and 2022, and the remaining 30% in 2023. The allocation of grants for 2021-2022 has been based on different socio-economic criteria.^12^

## Fiscal and Fiscal Policy Space: Can We Rebuild More Fairly?

 The pandemic brought the state back to support. Many countries responded with wage support, cash transfers, credit schemes, tax cuts and delays, support for importers and exporters, policy rate cuts, support for businesses, and subsidies or rent deferrals, that fueled speculation of a turning point in state/market dynamics, as Labonté puts it, reflecting the reality of what happened.

 The mechanisms imposed by governments and the support required in different areas underpin the need to create monetary and fiscal policies that are flexible, that are coordinated and that give the government the space to maneuver while navigating these enormous environmental and social challenges.^13^

 Including Labonté´s proposal, a single wealth tax could raise significant revenue and continue public support since this would not affect consumption and depend on income.

## Decrease/Post-growth: Should We Rebuild at All?

 The response of governments to the dramatic economic slowdown caused by the pandemic in the face of “normal” periods of recession usually involves cuts in public spending. However, the opposite has occurred in response to COVID-19, where unprecedented levels of public spending have been seen, resulting from political actions rather than economic facts.^14^ It is a parallel to be studied between degrowth and post-growth, the economic contraction induced by COVID-19 was involuntary and not a deliberative plan that emerged from a simple and large-scale democratic process, so it would be unfair to say that a planned transition to degrowth would have the same effects inequitable. The transition to a post-growth economy is, ultimately, a political decision that requires concrete policy options that favor the well-being of society rather than an endless expansion of gross domestic product.^15^ Moreover, one of the options for development is the option identified by Labonté, in which he indicates that a relatively new concept has entered the lexicon of environmental economics: degrowth, which captures the importance of reducing aggregate levels of global consumption to avoid the catastrophic collapse of ecosystems.

## Towards a Supportive Post-growth Economy: Can We Challenge the Rise of Autocratic Regimes?

 The transformation of capitalism through neoliberalism and achieving a sustainable solidarity economy requires governments willing to discipline markets for good public purposes and to initiate fiscal and tax policies that radically redistribute access to the resources people need for healthy living, confirming what Labonté states. The development of each of the countries must be seen in global markets led by the SDGs, with intermediaries who privilege the assumption of the principles of sustainable development and who assume the implementation of the Green New Deal as a duty.

## Conclusion

 The development of the pandemic has been a huge global event that has affected countries. Some countries were involved (developed countries) together with WHO for development, which was the proposed solution: the generation of the vaccine. Each of them has different dimensions, considering aspects of management, policies, and economic development. The measures taken by the governments to face the pandemic were presented by index; the public health index (considers 13 measures) and the index of economic measures (considers seven measures). Considering the indexes, it is possible to see that there is no direct relation between economic development, with the rigidity of the measures taken since there are countries belonging to the Organisation for Economic Co-operation and Development (OECD), and the level of rigidity is not the same for the group of countries belonging to the OECD.^16^

 The analysis developed makes it possible to identify the pandemic as a disruption in all kinds of aspects at a global level; Labonté´s focus on the global economic aspects of development and agreeing that the disruption from the health of people, disruption in markets. From every point of view, disruption in the way of governing, disruption in the economic models assumed or adopted by countries, a disruption from obtaining countries’ resources with their trade union policies and disruption that should make it possible to generate growth in through sustainable developments at a global level.

## Ethical issues

 Not applicable.

## Competing interests

 Author declares that she has no competing interests.

## Author’s contribution

 KCJ is the single author of the paper.
